# Reducing the causal illusion: a question of motivation or of information?

**DOI:** 10.1098/rsos.250082

**Published:** 2025-06-18

**Authors:** Aranzazu Vinas, Fernando Blanco, Helena Matute

**Affiliations:** ^1^ Department of Psychology, University of Deusto, Bilbao, Spain; ^2^ Department of Social Psychology, University of Granada, Granada, Spain; ^3^ Mind, Brain and Behavior Research Center (CIMCyC), Granada, Spain

**Keywords:** decision-making, cognitive bias, causal illusion, incentives, debiasing, causal judgements

## Abstract

The causal illusion is a cognitive bias that involves believing that one event causes another when it does not. It has negative consequences in different spheres of life, including health. Therefore, diverse interventions have been designed to reduce it. The more common ones are educational interventions. These include different elements related to improving both, motivation and information. We wanted to explore which of the two factors was more important for their effectiveness. We first used financial incentives to promote motivation (experiments 1a and 1b), but did not find them effective. Second, we used debiasing instructions about what has to be done to infer the causal relationship between two events accurately. This effectively reduced the causal illusion when the circumstances were in place for the illusion to be high (experiment 2). We discuss the results and their theoretical and practical implications.

## 1. Introduction

Causal thinking enables people to develop coherent narratives in which one event affects another. Thanks to this ability, people can explain and understand the world, reflect on their mistakes, make decisions and make predictions about the future [1,2]. Causal thinking is such a helpful device to adapt to our environment that we often overuse it. This is illustrated by a phenomenon called causal illusion, a cognitive bias that produces the belief that one event causes another when, in fact, no such connection exists. This cognitive bias has positive consequences, like protection from learned helplessness [3,4]. It is also related to reduced levels of anxiety and depressive symptoms [5,6], better emotion regulation and improved mental health [7]. However, above all, the causal illusion has negative consequences because it is associated with mistaken beliefs, polarized attitudes, ideological extremism, prejudices and social stereotypes [8,9]. Additionally, it has been related to superstitions, irrational beliefs [9] and pseudomedicine usage [10–12], the latter being one of the domains in which more research on causal illusions has been conducted. Pseudomedicines are medical treatments that lack scientific evidence. Examples include homoeopathy [13], or dietary supplements against dementia [14] among many others. These pseudomedicines can have negative health consequences, including side effects from unnecessary treatments, additional financial costs and opportunity costs resulting from delays or forgoing effective treatments [11,15,16].

In order to infer correctly the causal relationship between a potential cause and its potential effect, people need to consider the probability of occurrence of the potential effect in the presence and in the absence of the cause and compute the contingency between the two events. When the difference between these two probabilities is zero, the contingency between the two events is null, which suggests that there is no causal relationship between them. However, as previously mentioned, under certain circumstances people tend to overestimate causal relationships, showing an illusion of causality.

Although much research has been conducted on what factors influence the overestimation of causal relationships, the mechanisms that underlie this bias are still debated. In this regard, there are broadly two main approaches. First, some researchers assume that the illusion is a *learning effect*, which means that it can be traced down to how contingency information is encoded during the experiment. For example, people would pay more attention to, or encode more easily, those cases where the potential cause is present rather than those where it is absent. As a consequence, the information that they have available when they need to compute the contingency between the two events would be biased, and hence the overestimation of causality. Alternatively, other researchers propose that the illusion is a *judgement effect*. According to this view, the illusion would be formed at the moment of making the judgement, after the relevant information has been encoded. Thus, according to this second view, people acquire and encode the information correctly but do not use it properly, giving more importance to certain cases, particularly those in which the potential cause is present rather than cases where the potential cause is absent (for a review see [17]).

Regardless of the actual mechanism, because of the drawbacks associated with the causal illusion, and specifically with pseudomedicine usage, previous research has aimed to reduce this bias. The usual approach is through educational interventions that have proven to be effective for debiasing (e.g. [11,18–21]; see [22] for long-lasting results of a large-scale intervention). They usually consist of face-to-face sessions, lasting about 1 h, in which an expert explains to a group of participants what the causal illusion is, warns them that it is a pervasive bias, and instructs them on what to do to avoid it. That is, the instructor explains to them that they should sample and compare both potential cause-present and potential cause-absent trials, in order to achieve more accurate judgements.

We asked ourselves which elements included in the existing educational or debiasing interventions contributed to a greater extent to reduce the causal illusion. In particular, we focused on two different types of components. On the one hand, Chow *et al*. [20] found that a debiasing instruction consisting on instructing on the scientific method reduced (but did not eliminate) the causal illusion. Similarly, we wanted to test whether the debiasing instruction usually used in educational interventions would suffice to reduce the illusion. On the other hand, it is also possible that those elements linked to the participants’ motivation could also be important in successful interventions. It could be the case that, because judging causality is effortful, if people lack internal motivation they might not gather the necessary information and/or make the necessary cognitive effort to combine the information to produce accurate judgements. To this end, we incentivized participants economically, rewarding their correct causal judgements. This approach is common in certain research areas aiming to shape behaviours [23–25], but has not been previously tested in interventions aiming to reduce the causal illusion.

If the causal illusion is generated in the post-acquisition judgement phase, then the financial incentive would work to improve the accuracy of judgements even if it is presented at the time when the judgement is requested. On the contrary, if the causal illusion is a learning effect, then the incentive should only work to improve accuracy if it is presented before the learning phase begins, then the incentive would shape the behaviour during the learning task, encouraging participants to collect more representative information, including both types of trials, when the cause is present and when the cause is absent. To answer this question, we introduced the incentive in the judgement phase (experiment 1a) and then before the learning phase (experiment 1b).

Moreover, the causal illusion is a pervasive cognitive bias that is difficult to eliminate [20], particularly when the expected effect occurs frequently [26,27]. In addition, the illusion is also high when the probability of occurrence of the potential cause, P(C), is high as well, perhaps because this facilitates paying more attention or giving more weight to the trials in which the potential cause and the effect co-occur than trials in which they do not [26,28,29]. In these situations, the causal illusion tends to be strong. Because of that, it is particularly interesting to test interventions to reduce the illusion in situations like these ones, associated with stronger biases. Particularly interesting are those settings in which the participants themselves are the ones responsible for deciding whether to introduce the potential cause in each trial (i.e. active tasks) because, if they are trying to obtain the effect, they often tend to introduce the cause in a high percentage of trials [30,31], and this increases the illusion. Thus, the probability with which participants introduce the potential cause, P(C), often mediates the development of the illusion [18,32,33]. In this regard, Vinas *et al*. [34] recently found that the causal illusion can also be especially high when not only the effect is frequent, and the task is active, but also, when abundance of resources exists. This happens because, in active tasks, if the resources are abundant, participants tend to introduce the cause in many (and even all) trials, thereby they tend to oversample trials in which the cause is present relative to the trials in which it is absent, so their P(C) mediates the development of the illusion.

Thus, we proposed to examine the effect of the two components of the educational interventions (either instruction-based or motivation-based) in two different contexts, one that usually leads to strong illusions (abundant resources) and one that shows smaller illusions (shortage of resources). To sum up, we designed our experiments intending to isolate each of these elements (motivation and information) and explore whether they influenced the causal illusion in situations that are known to be more and less prone to it. Thus, in experiments 1a and 1b, we tested the effect of financial incentives to increase accuracy, before moving to an instructional debiasing intervention in experiment 2.

## 2. Experiment 1a

The experiment manipulated two factors: the availability of resources during the simulated task and the presence of incentives for accurate judgements. Financial incentives are a particular form of manipulating motivation that has been promising in other domains [35–39]. We expected that both the available budget and the incentive would influence the causal illusion so that participants in the scarcity condition and those with financial incentives would show lower causal illusions.

### 2.1. Methods

#### 2.1.1. Participants

We conducted a sensitivity analysis before data collection. With a sample of 200 participants, which was decided for economic and practical reasons, we could detect a medium effect size, *f* = 0.22, with 80% power in the 2 × 2 interaction. Therefore, we recruited this number of anonymous participants through the UK-based online platform Prolific Academic (distribution by gender: 112 women, 82 men and six people self-defined as other; age: *M* = 28.0, s.d. = 8.25). The computer program randomly assigned them to the experimental groups (distribution by group: scarce *n* = 52, wealthy *n* = 61, scarce-incentive *n* = 45, wealthy-incentive *n* = 42). We used filters to limit the sample to people speaking fluent English who had not participated in previous experiments conducted by our research group. We included an attention check (since all of our participants answered it correctly, we did not exclude any). We estimated 10 min to complete the experiment and paid all participants £1 for their time. Additionally, we offered a maximum £2 incentive to participants in groups with incentives (scarce-incentive and wealthy-incentive). The study pre-registration is available at https://aspredicted.org/uh89g.pdf.

#### 2.1.2. Procedure

We adapted the task from Vinas *et al*. [34] to study how economic constraints influence the causal illusion. At the beginning of the experiment, participants read the instructions: we asked them to imagine they were doctors treating a rare (fictitious) disease with a (fictitious) treatment. Importantly, we advised them that the treatment was still under development, and its effectiveness was not yet proven. We then asked participants to heal as many patients as possible and, at the same time, manage the budget efficiently. Additionally, we told the participants either that the budget available to buy the treatment was high and that ‘there was usually a surplus’ (wealthy groups) or that it was very tight and ‘usually run out’ (scarce groups), hence implementing the resource availability manipulation.

After reading the instructions, the participants proceeded to the learning phase, which comprised 30 trials. Each trial portrayed an individual patient, and the participant had to choose whether to administer the treatment to that patient. To reinforce the budget manipulation, participants constantly saw a reminder of the available budget (scarce or abundant) and a budget bar at the top of the screen. With each treatment dose used, the budget bar was updated by displaying a reduction. Consistent with the instructions, this reduction was larger in the scarce groups: each dose administered reduced the bar by 1/30, compared with 1/300 in the wealthy groups. In fact, despite the difference in instructions, the resources were enough to buy doses for all patients in all the groups.

One of our main dependent variables was the probability with which the participants used the treatment, P(C). To calculate it, we took the number of doses the participant administered during the whole session and divided it by the total number of trials (i.e. 30 patients). Immediately after the participant decided whether to treat a patient, the next screen informed whether the patient healed or not. Importantly, unbeknownst to the participants, the same percentage of patients healed when they received the treatment as when they did not (70%), so the contingency was zero. That is, the probability of the potential effect was high and the treatment was a pseudomedicine (because using it did not increase the probability of healing above the base rate, i.e. using no treatment). This is a typical situation in experiments investigating the causal illusion [27].

Once the participants had visited all 30 patients, they proceeded to the testing phase in which we measured their causal illusion through a numeric judgement. They had to answer the following question: ‘To what extent do you think the Batatrim medicine is effective in curing the Lindsay Syndrome crisis?’, on a scale from zero (completely ineffective) to 50 (moderately effective) to 100 (completely effective). It is also possible to use bidirectional scales (i.e. from −100 to 100) in research on causal illusion (see [40] for a review). In fact, when bidirectional scales are used, causal judgements are smaller [40]. It might be that unidirectional scales have an anchoring effect around 50, but it might also be that bidirectional scales have an anchoring effect around 0, and that they underestimate the causal illusion as a consequence. Anyway, we decided to use a unidirectional scale for the sake of consistency with previous studies [34]. Moreover, we used the same unidirectional scale in all groups in all three experiments (1a, 1b and 2). Because the same percentage of patients healed regardless of whether they received the treatment, the correct answer was zero. Nonetheless, although the programmed contingency was zero, given that in this type of task, the participants had to choose whether to give the treatment to each patient, there could in principle occur deviations in the actual experienced contingency [18]. These deviations are normally small in magnitude and random [30]; therefore, they cannot fully explain the results (see the experienced contingency per group in the electronic supplementary material).

Just before the judgement phase, and only in the case of groups with incentives (scarce-incentive and wealthy-incentive), we informed these participants that we would give them a maximum £2 incentive if they answered correctly (they would start at £2 that would be discounted at a rate of 2 cents for each point in the scale). Consequently, these participants obtained a bonus depending on how much their answers departed from the correct value of zero.

### 2.2. Results and discussion

#### 2.2.1. Influence of budget and incentives on P(C)

**Figure 1.**
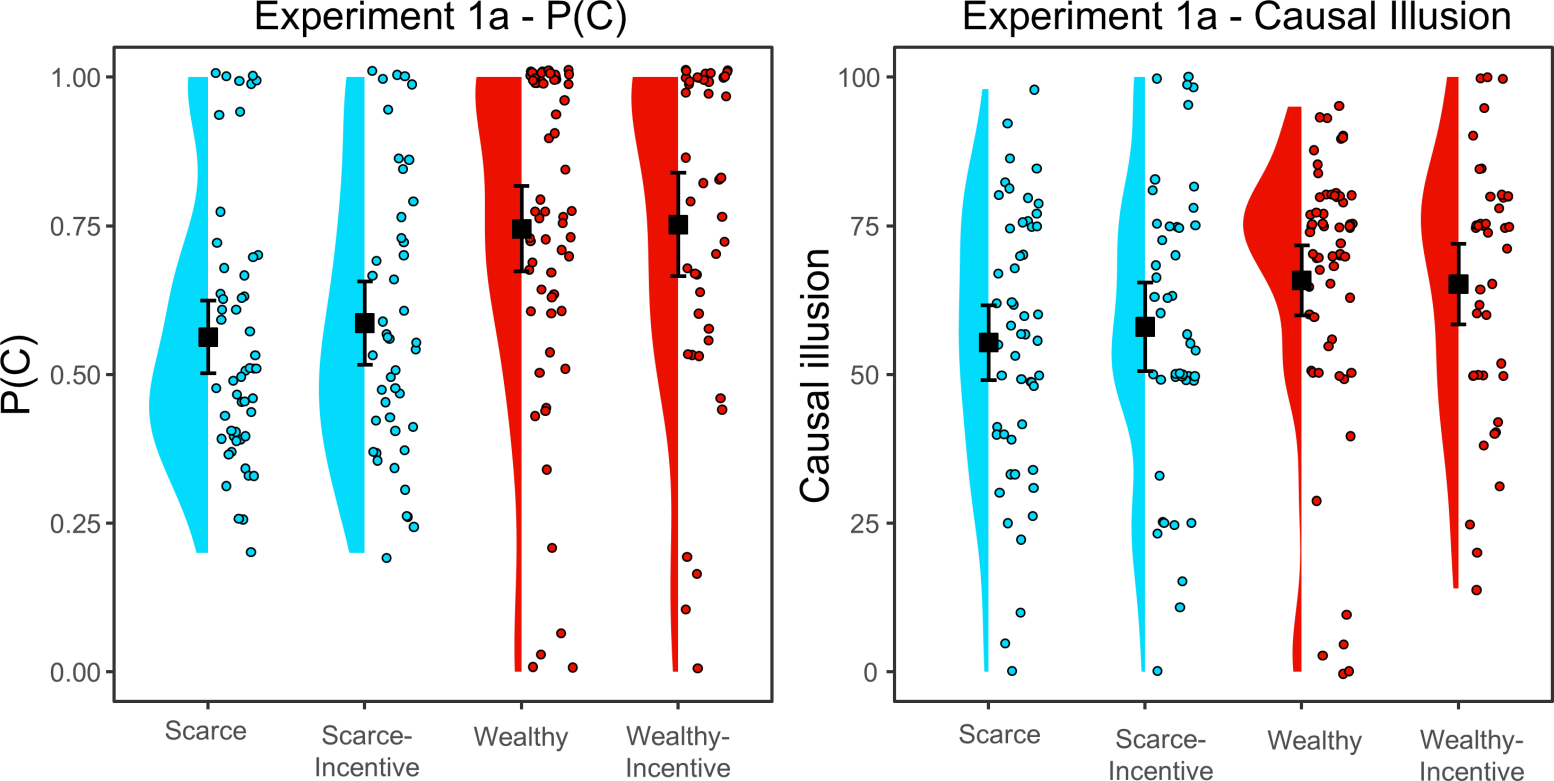
P(C) and causal illusion per group in experiment 1a. Black squares depict group means, and error bars depict 95% confidence intervals for the mean.

Figure 1 (left panel) shows the P(C) (proportion of treated patients) in each group. Since the incentive was given after the learning phase, immediately before the judgement, we did not expect that incentives would influence P(C). On the other hand, the budget manipulation should have an effect. To test this, we performed a 2 × 2 ANOVA (budget [scarce, wealthy] × incentive [yes, no]) on P(C). The results showed a main effect of budget, *F*(1, 196) = 22.77, *p* < 0.001, *η*^2^ = 0.104, such that participants with a scarce budget administered significantly fewer pseudomedicine doses than participants with abundance. As expected for the P(C), neither the effect of incentive nor the interaction was significant, both *F*s < 1.

#### 2.2.2. Influence of budget and incentives on causal illusion

Then we analysed the causal illusion (see figure 1, right panel) by conducting a 2 × 2 ANOVA (budget [scarce, wealthy] × incentive [yes, no]). As with P(C), the results also showed a main effect of the budget on causal illusion, *F*(1,195) = 23.946, *p* = 0.008, *η*^2^ = 0.035, so a wealthy budget promotes stronger illusions. However, we did not find a main effect of the incentive, *F*(1, 195) = 0.268, *p* = 0.605; and the interaction between budget and incentive was also non-significant, *F*(1,195) = 0.312, *p* = 0.577. Thus, we conclude that the budget influenced the causal illusion as expected: participants with scarcity exhibited significantly lower illusions than wealthy participants. In contrast, the financial incentive offered before the testing phase did not seem to influence the judgements. To determine whether the data supported the null hypothesis for the effect of the incentive on the causal illusion, we computed a Bayes factor using the default *a priori* distribution recommended by the Jamovi program, a Cauchy with *r* = 0.707. The Bayes factor in favour of the null hypothesis was *BF_01_* = 6.38 (*%error* = 4.08 × 10^−6^). This is interpreted as moderate evidence in favour of incentives not influencing the causal illusion.

Previous research has consistently shown that the higher the P(C), the greater the causal illusion [26,41]. If participants introduce the potential cause in a high percentage of trials, they sample proportionally fewer instances in which the effect occurs in the absence of the cause. Thus, the information they have when they compute the contingency is already biased, and this favours the development of the illusion. Reducing the P(C) so that participants can be equally exposed to trials in which the cause is present and trials in which the cause is absent has been considered a potential mechanism for interventions to reduce the bias [18].

Thus, once we had documented the effect of the budget on causal illusion, we proposed (also in line with previous research, see, for example [34], that this effect could be mediated through P(C). Therefore, we collapsed the groups with the same budget (scarce and scarce incentive on the one hand, and wealthy and wealthy incentive on the other) and conducted a mediational analysis. The results confirmed that the total effect of the budget on causal illusion was significant, *Z* = 2.777, *β* = 0.193, *p* = 0.005. We also found that the indirect effect of the budget on causal illusion through P(C) was significant, *Z* = 4.871, *β* = 0.326, *p* < 0.001, whereas the direct effect was not, *Z* = −0.124, *β* = −0.007, *p* = 0.902, which indicates complete mediation. That is, the limited budget reduced the amount of pseudomedicine administered and this, in turn, seemed to reduce the causal illusion. One has to consider, however, the limitations of interpreting mediational analyses in this type of research (e.g. uncontrolled confounding factors, etc.).

## 3. Experiment 1b

The previous experiment showed that the financial incentive did not affect causal illusion. In contrast, the budget restriction effectively reduced the illusion through the P(C). Therefore, in experiment 1b, we aimed to test whether informing participants about the existence of the financial incentive before, rather than after, the learning phase, could be more effective in reducing the causal illusion. We thought that providing this information in advance would help participants to (i) collect all the necessary information during the experiment (i.e. to sample both cause-present and cause-absent trials), and (ii) to encode both types of information correctly, which should eventually help them improve the accuracy of their causal judgement.

### 3.1. Methods

#### 3.1.1. Participants

As in the previous study, we aimed to recruit 200 participants from Prolific Academic. Due to an error in the database dump, the data from one participant was lost, so the final sample was *n* = 199 (distribution by gender: 109 women, 87 men and three people self-defined as other; age: *M* = 29.7, s.d. = 7.99; distribution by group: scarce *n* = 41, wealthy *n* = 53, scarce-incentive *n* = 52, wealthy-incentive *n* = 53). As in experiment 1a, we used filters to limit the sample to people speaking fluent English and who had not participated in previous experiments conducted by our research group. We did not exclude any participants (all of them correctly answered to the attention check). We paid the same amount as in experiment 1a: £1 for their time to all the participants, plus a maximum £2 incentive to participants in the groups with incentives (scarce-incentive and wealthy-incentive). The study pre-registration is available at https://aspredicted.org/8as2s.pdf.

#### 3.1.2. Procedure

The procedure was identical to the one in experiment 1a, except for an important addition in the instructions. Immediately before the learning phase, we advised all participants that at the end of the experiment, we would ask them to answer the question ‘To what extent do you think the Batatrim medicine is effective in curing the Lindsay Syndrome crisis?’ , on a scale from zero (completely ineffective) to 50 (moderately effective) to 100 (completely effective). Additionally, on the same screen, we informed participants in the groups with incentives (scarce-incentive and wealthy-incentive) that if they answered correctly, they would receive a maximum £2 incentive (in the same conditions as experiment 1a). Thus, participants knew from the beginning what question they would have to answer at the end of the task and how their accuracy would be incentivized.

### 3.2. Results and discussion

#### 3.2.1. Influence of budget and incentives on P(C)

**Figure 2.**
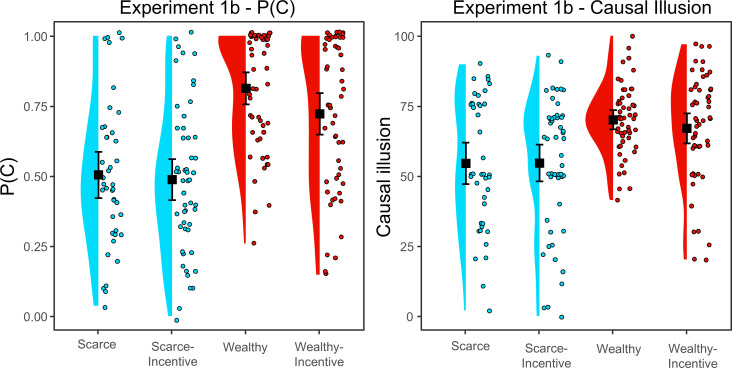
P(C) and causal illusion per group in experiment 1b. Black squares depict group means and error bars depict 95% confidence intervals for the mean.

Figure 2 (left panel) shows the mean P(C) in each group. We expected the budget to influence the P(C), as we had found in experiment 1a. Additionally, since the incentive was reported before the learning phase, in this case, we also expected the incentive to influence the P(C), perhaps by producing values closer to 0.50 (i.e. similar amounts of treated and not treated patients to test better the efficacy of the treatment). To test our hypotheses, we conducted a 2 × 2 ANOVA (budget [scarce, wealthy] × incentive: [yes, no]) on the P(C). The results showed a significant main effect of budget, *F*(1, 195) = 58.11, *p* < 0.001, *η*^2^ = 0.227. However, we did not find a main effect of incentive, *F*(1, 195) = 2.31, *p* = 0.130, and the interaction was non-significant, *F*(1, 195) = 1.09, *p* = 0.298. We obtained a Bayes factor for the effect of incentive on P(C), which proved inconclusive: *BF_01_* = 1.42, % error = 2.20 × 10^−6^. That is, as in experiment 1a, participants with scarcity administered significantly less pseudomedicine than those with abundance, but we did not find evidence that the incentive presented before the learning phase influenced the pseudomedicine usage.

Finally, we ran an additional (non-pre-registered) analysis to check whether the frequency with which participants administered the treatment was lower in experiment 1b compared with experiment 1a, that is, that P(C) depended on the moment in which we informed about the existence of the incentive. However, we did not find significant differences in P(C) between the two experiments (see electronic supplementary material), suggesting that financial incentives did not affect the decision to use the treatment during the task.

#### 3.2.2. Influence of budget and incentives on causal illusion

The results concerning causal illusion appear in figure 2, right panel. To test whether both budget and incentive influenced causal illusion, we conducted a 2 × 2 ANOVA (budget [scarce, wealthy] × incentive: [yes, no]) on this variable. As with P(C), the results also showed a main effect of the budget on causal illusion in the expected direction, *F*(1,195) = 23.946, *p* < 0.001, *η*^2^ = 0.109. However, neither the main effect of the incentive, *F*(1, 195) = 0.268, *p* = 0.605, nor the interaction were significant, *F*(1,195) = 0.312, *p* = 0.577. The Bayes factor in favour of the null hypothesis that the incentive did not produce differences in causal illusion was *BF_01_* = 4.78 *(*% error = 3.01 × 10^−6^), which is moderate evidence in favour of the null hypothesis. This suggests that the incentives did not work to reduce the causal illusion.

Finally, for consistency with the previous experiment, we tested whether the relationship between causal illusion and budget was mediated by P(C). Thus, we performed a mediational analysis that included all four groups. The results confirmed that the total effect of the budget on causal illusion was significant, *Z* = −4.939, *β* = −0.331, *p* < 0.001. In addition, the indirect effect of the budget on causal illusion through P(C) was also significant, *Z* = −5.861, *β* = −0.278, *p* < 0.001, whereas the direct effect was not, *Z* = −0.833, *β* = −0.053, *p* = 0.405. That is, as in experiment 1a and also in line with the findings in Vinas *et al*. [34], the relationship between causal illusion and budget was completely mediated by P(C). In short, once again, we found that the amount of pseudomedicine administered mediated the relationship between budget and causal illusion, so that participants with scarce budget administered the treatment less often, and this fact seemed to be the cause of the reduction of the causal illusion.

However, based on the non-significant differences between groups, we cannot assure that financial incentives can reduce causal illusions even when presented before the learning phase. Nevertheless, we believe that our results provide additional evidence to the debate on whether financial incentives are indispensable in experimentation, both in Psychology and in Economics (see [23–25,42,43]; and on whether they should be reported at the beginning of the experiment and in what kind of tasks their use may be interesting [42].

## 4. Experiment 2

The results of experiments 1a and 1b indicate that financial incentives, either provided at the beginning of the learning phase or right before the judgement phase, seem unable to reduce the causal illusions that participants tend to show especially in situations of abundant resources. Consequently, we decided to test whether providing debiasing instructions would be useful for reducing the causal illusion. To this end, we based on the instructional manipulation previously used by Matute [31]. The idea in Matute's study was to specify, through instructions, slightly different goals for the participants, and those instructed to find out how effective the cause was, received additional information on how they should best do it. Thus, ‘naturalistic’ participants were instructed to try to obtain the desired effect (which in Matute’s work was a flashlight, and in ours, the healing) as often as possible. It is assumed that, in general, people spontaneously adopt this approach, which eventually would lead to higher responding rates, P(C), which is known to increase the illusion (as our mediational analyses revealed above) because participants get only exposed to information on cause-present trials and do not know what happens when the cause is absent. By contrast, ‘analytic’ participants were asked to assess accurately the strength of the causal link in the task (which in our procedure would be to find out how effective the treatment is). This goal was accompanied by the advice to sample as many cause-present trials as cause-absent trials, to obtain a balanced set of information. Thus, adopting the analytic stance leads to a P(C) closer to 0.50, and, consequently, to a weaker illusion because participants test what happens when the cause is present, as well as when it is absent. Although the distinction between analytic and naturalistic instructions was not designed as a debiasing intervention, the information contained in the naturalistic instructions is presumably what participants do by default, and the analytic instructions coincide with the information usually presented to participants in the educational interventions aiming to reduce the causal illusion (for example [18]).

In sum, in experiment 2, orthogonal to the budget manipulation, we used two different sets of instructions. According to these instructions, the participants’ goal was either to heal as many patients as possible or to assess the effectiveness of the treatment, and in the case of participants who had to evaluate the effectiveness of the treatment, we instructed them on how to do so. That is, we advised them that the causal illusion is a common bias and that, to achieve their goal, they needed to collect information about the probability of healing when they administered the treatment and also when they did not. Importantly, when writing this instruction, we were inspired by the information usually transmitted to the participants in the educational interventions. Consistent with the findings of Chow *et al*. [20], who instructed the participants to compare the treatment effects when the treatment was present versus when it was absent, we expected this information to effectively reduce bias. We especially expected it to help wealthy participants, as they tended to show a stronger causal illusion.

### 4.1. Methods

#### 4.1.1. Participants

The sample consisted of 200 participants from Prolific Academic (distribution by gender: 67 women, 130 men and three people self-defined as other; age: *M* = 29.2, s.d. = 10.2; distribution by group: scarce *n* = 50, wealthy *n* = 51, scarce-debiasing *n* = 53, wealthy-debiasing *n* = 46). Only English fluent speakers who had not participated in previous experiments conducted by our research group were admitted. We paid £1 for their time to all participants. We also pre-registered this study (see https://aspredicted.org/2z3ru.pdf).

#### 4.1.2. Procedure

**Figure 3.**
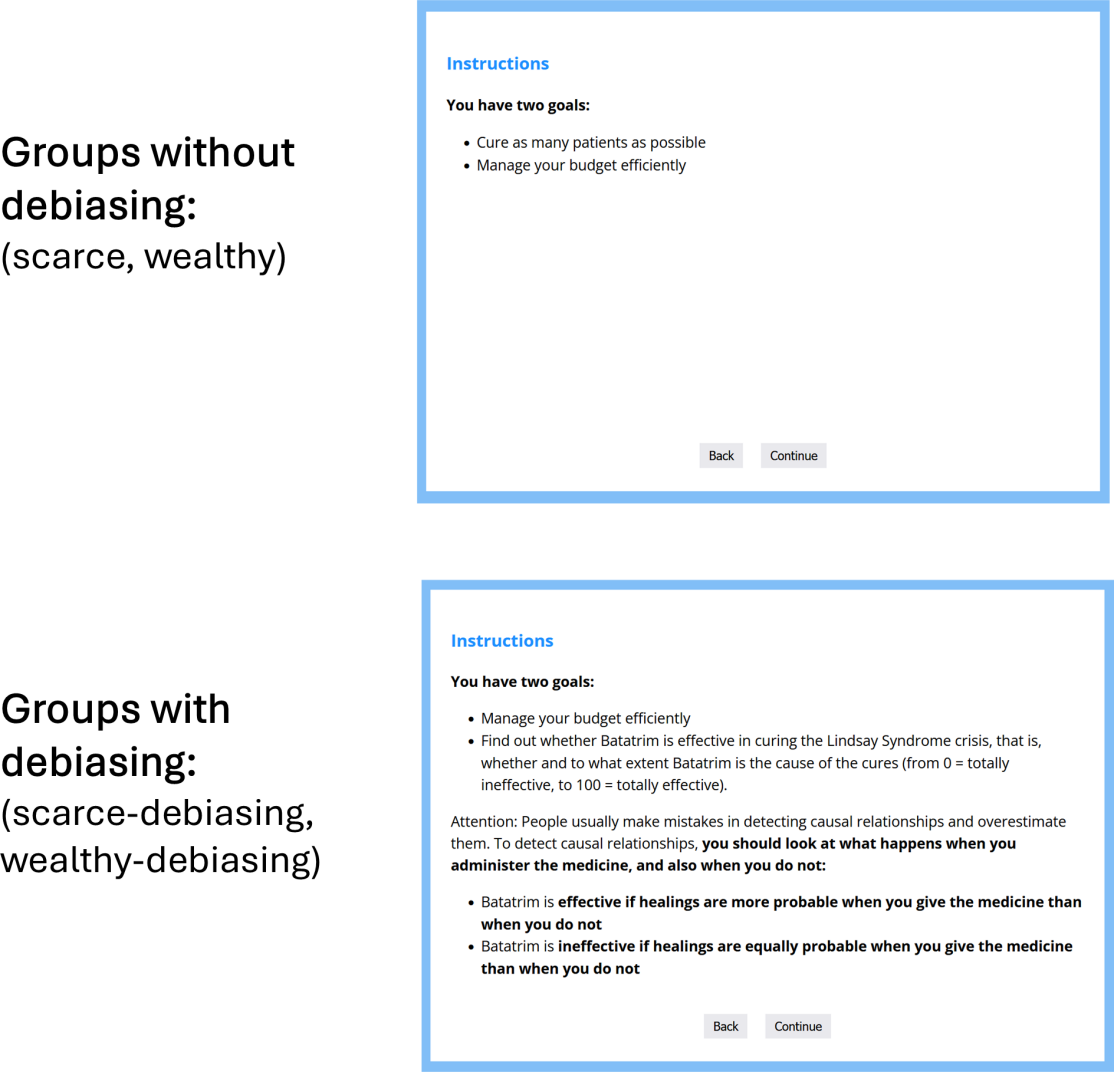
Experiment 2 instructions: task goals per group.

The procedure was similar to that of experiments 1a and 1b. We only modified the instructions as follows. First, we removed the references to financial incentives for correctly responding. Second, on the screen describing the task goals, the scarce and wealthy groups saw the same text as in the previous experiments, describing the two goals of the task (‘Cure as many patients as possible’ and ‘Manage the budget efficiently’, see figure 3, top panel). However, participants in the two groups with debiasing instruction (scarce-debiasing and wealthy-debiasing) read the modified version depicted in the bottom panel of figure 3. This instruction stated that the goals were to manage the budget efficiently and to find out whether the treatment was effective (i.e. an analytic instruction). Additionally, they read an explanation about the typical mistake that people often make when judging causality, and how to avoid it by comparing the probability of healing when the treatment is taken (i.e. cause-present trials) against the probability of healing when the treatment is not taken (cause-absent trials), so that, if the two probabilities are identical, then one should conclude that the treatment is not working. Different from Matute [31], no explicit instruction was given as to whether one should keep a P(C) close to 0.50 to assess the effectiveness of the treatment correctly, but it was expected that participants would do it by themselves.

### 4.2. Results and discussion

#### 4.2.1. Influence of budget and debiasing instruction on P(C)

**Figure 4.**
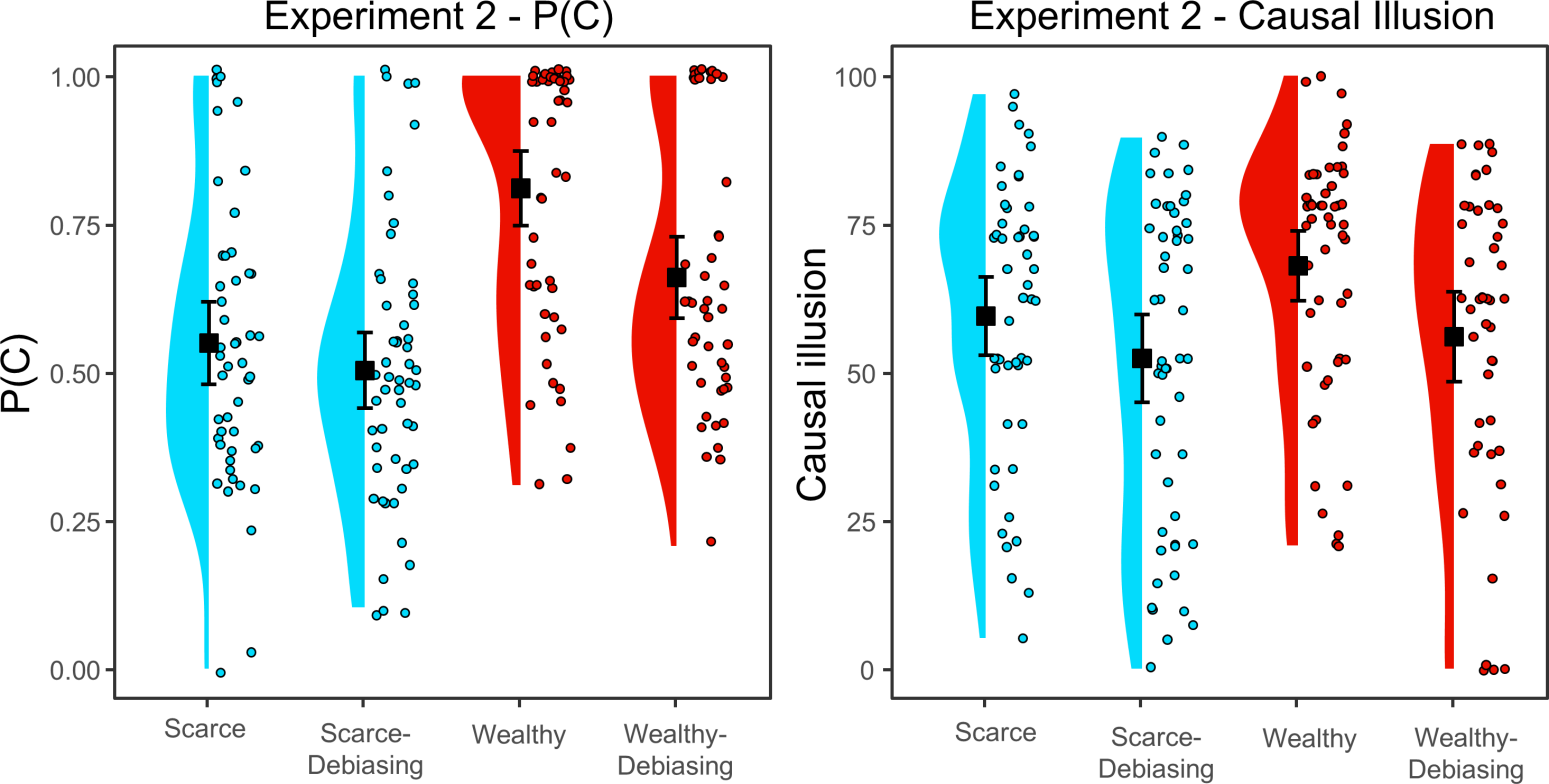
P(C) and causal illusion per group in experiment 2 . Black squares depict group means, and error bars depict 95% confidence intervals for the mean.

Although the instructional manipulation (analytic versus naturalistic) in Matute’s study [31] indicated that participants in the analytic condition should reduce their P(C) to get a balanced sample of information, in our experiment, the indication was not explicit: the importance of comparing cause-present and cause-absent trials was made, but without explicit instruction to reduce P(C). Still, we expected that the debiasing instruction should lower P(C) to a value closer to 0.5, particularly in the wealthy budget condition. Thus, the first analysis was a 2 × 2 ANOVA test (budget [scarce, wealthy] × debiasing instruction [no, yes]) on the P(C). The results (portrayed in figure 4, left panel) showed a significant main effect of the budget, *F*(1, 196) = 39.90, *p* < 0.001, *η*^2^ = 0.161 and a significant main effect of the debiasing instruction, *F*(1, 196) = 8.82, *p* = 0.003, *η*^2^ = 0.036. However, the interaction between budget and debiasing instruction was non-significant, *F*(1,196) = 2.48, *p* = 0.117. As expected, post hoc contrasts showed significant differences between the wealthy and wealthy-debiasing groups, *t*(1, 196) = 3.17, *p* = 0.002. However, also as expected, although the scarce-debiasing group administered somewhat less treatment, the difference in P(C) between the scarce and scarce-debiasing groups was non-significant, *t*(1, 196) = 1.00, *p* = 0.318. This is probably because participants with abundant resources administered significantly more pseudomedicine than those facing scarcity and hence, the effect of the debiasing instruction is more effective in the abundant condition than in the scarce one because more room for improvement exists. These results replicate previous experiments and suggest that the budget effect on P(C) is robust. Furthermore, as we expected, the debiasing instruction reduced the amount of pseudomedicine administered, although this was only significant in the wealthy condition.

#### 4.2.2. Influence of budget and debiasing instruction on causal illusion

Then we tested the same predictions on the causal illusion using a 2 × 2 ANOVA test (budget [scarce, wealthy] × debiasing instruction [no, yes]) (see figure 4, right panel). Contrary to our expectations, we did not find a main effect of budget, *F*(1, 196) = 3.145, *p* = 0.078. However, a Bayes factor analysis of the effect of the budget was inconclusive, *BF_01_* = 1.283 (% error = 1.821 × 10^−6^). On the other hand, the effect of debiasing instruction was significant, *F*(1,196) = 7.756, *p* = 0.006, *η*^2^ = 0.037. When it was incorporated, participants tended to show lower causal illusions than when it was not. Finally, the interaction between budget and debiasing instruction was non-significant, *F*(1,196) = 0.490, *p* = 0.485. However, post hoc contrasts showed significant differences between the wealthy and wealthy-debiasing groups, *t*(196) = 2.427, *p* = 0.016, although the difference between the scarce and scarce-debiasing groups was non-significant, *t*(196) = 1.498, *p* = 0.136. That is, participants in the wealthy-debiasing group showed a lower causal illusion than those in the wealthy group, but this did not happen in the case of participants with scarcity. Probably they did not have as much room for improvement as participants with abundance: their causal judgement was already lower and the debiasing instructions could not reduce it further.

As we have mentioned, it is proposed that the reduction in P(C) is one of the main mechanisms by which the analytical debiasing instructions work [31]. Therefore, given that we found a main effect of our debiasing instruction on causal illusion, we conducted a mediation analysis on the whole dataset (collapsing across budget levels) to examine whether this effect was mediated by P(C). The results confirmed that the total effect of the debiasing instruction on causal illusion was significant, *Z* = −2.83, *β* = −0.197, *p* = 0.005. We also found that the indirect effect of the instruction on causal illusion through P(C) was significant, *Z* = 9.04, *β* = 0.539, *p* < 0.001, while the direct effect was not, *Z* = −1.48, *β* = −0.088, *p* = 0.139. This indicates that the mediation was complete and, hence, that the debiasing instruction produced a lower frequency of pseudomedicine use which, in turn, seemed to translate into a lower causal illusion, as we expected.

## 5. General discussion

The present research aimed to explore which elements of the educational interventions usually used to reduce the causal illusion are more effective, especially when this cognitive bias is expected to be high. Thus, we examined two different options: (a) financial incentives, which are usually used for promoting motivation in experiments and real life, and (b) presenting instructions that are useful for debiasing.

Financial incentives are routinely used in economics and psychology, although with different aims and focus. Behavioural economists are usually interested in how people’s decisions adapt to incentive structures by evaluating costs and benefits. Consequently, if the incentives for a response are unclear, the experiment is considered uninterpretable [44]. That is why most experiments in economics tend to transparently communicate the payoff matrix to participants, and try to provide motivating incentives (generally, in the form of payment). This contrasts with the standard practice in psychology, where the focus tends to be on processes and it is assumed that incentives in real life are often unclear. Here, in the context of a study of psychological processes, we incorporate financial incentives to increase motivation for accuracy in experiments 1a and 1b. Concerning this question, there is an ongoing debate in psychology and behavioural economics. On the one hand, some research works suggest that incentives improve performance in a variety of scenarios (e.g. better performance at the workplace [37], less biased online reviews [38], improvement in certain probabilistic reasoning tasks [35], improved accuracy in rapid stimulus categorization [36], and reduced anchoring bias [39]). On the other hand, others propose that the effect of incentives is unclear, revealing several factors that can modulate it. For instance, Weibel *et al*. [45] propose that, for inherently interesting tasks, incentives can hamper, rather than improve, performance. Similarly, the review by Bonner *et al*. [46] suggests that complex tasks usually do not benefit from incentives. Read [43] concludes that incentives are unnecessary to achieve good performance and even advocates for not requiring them to conduct experiments in behavioural economics. Given this mixed evidence, it is not surprising that our two experiments fail to detect any advantage of financial incentives to reduce the causal illusion.

One might argue that our incentive was too low to work, but we believe this is not the case. Previous literature insists that to be effective, financial incentives must be sufficiently high [23,42]. That is why we set a £2 incentive (in addition to the £1 payment for participating in the experiment) if participants responded correctly to the causal judgement, that is, a participant could receive up to £3 (which would be the equivalent of £18 per hour). We decided on this amount for two reasons: this was a high payment for the average amount usually paid to Prolific Academic participants, which is between £6 and £8 per hour (https://www.prolific.com/participants); and the incentive represented a high increase in the payment that participants expected when they accepted to participate in the experiment (£1). That is, the relative incentive was high, and it is well known that the relative value of incentives is more important than their absolute value on motivation (see [47–49]).

A second point that deserves discussion concerning our incentive manipulation is that we tested two different moments to introduce it: either before the learning phase (experiment 1b), or after it (i.e. just before the judgement, experiment 1a). We believe that the choice was important because it has implications for the mechanisms underlying the causal illusion. Thus, as we advanced above, two approaches have been described in the literature: some propose that the illusion is a cognitive bias that appears at the moment of collecting and encoding the information (a ‘learning’ bias), while others argue that it manifests when the judgement is requested at a subsequent time, perhaps as a consequence of how the previously encoded information is combined or analysed (i.e. a ‘response’ bias; see [17] for a review). The straightforward prediction is that, if the former proposal is correct, incentives should only work to improve judgement when they are presented before the learning phase. However, we found no clear evidence for the effect of incentives in either moment (neither in experiment 1a nor in experiment 1b).

A possible limitation of our study is that the goals we set for the participants could be understood as motivation elements by themselves. We intended the goals to be as realistic as possible (either to heal as many patients as possible or to find out the effects of the treatment, while obviously taking care of the available budget in all cases). Therefore, motivation (albeit not financial) and information would be confounded in experiment 2. We did so in an attempt to reproduce the conditions of debiasing interventions, and our results effectively show that yes, setting an analytic mode of thinking as a goal (analysing the effectiveness of the treatment) while instructing participants on how to achieve such goal, did reduce the illusion that develops when no such goal (and instructions on how to achieve it) are provided. Nonetheless, it would be interesting to disentangle in future experiments whether the analytic goal and the instruction on how to achieve it are both necessary and/or sufficient components of the debiasing interventions.

Another important feature of our study is that we wanted to explore how we could reduce the causal illusion in a situation in which it was expected to be high. That is why we introduced a budget manipulation. Moreover, to model a more realistic situation, as mentioned, we also introduced competing (but realistic) goals (i.e. healing as many patients as possible and managing the budget efficiently). These two factors (the budget manipulation and the goal instruction) could have overshadowed the effect of incentives on the quantity of treatment administration. In this sense, as a line of future research, we believe that it would be interesting to design simpler experiments, in which fewer factors were manipulated, to detect which ones are critical. Relatedly, it could also be interesting to control the P(C) to separate the effects of P(C) mediation on the causal judgements (see an example in [32]).

In any case, given that financial incentives did not yield a conclusive result, and although we cannot say for sure, it seems that reducing the causal illusion is not related to external financial motivational elements for fostering people to give correct judgements, or at least that this is not the only factor that influences it. This might be an indication that participants generate the causal judgement through a process that they believe is appropriate, hence, financial incentives do not convince them to change their strategy. That is why we tried a different approach in experiment 2. In the interdisciplinary field of decision-making, it is acknowledged that people’s decisions are subject to several systematic errors or cognitive biases, particularly when these are made without much reflection and are driven by intuition [50]. Therefore, different debiasing strategies have been designed to counter cognitive biases [51], often with promising results [52]. Most debiasing interventions revolve around providing education to make the individuals aware of their own bias.

Although the effectiveness of debasing is not clear (see some failures in [53,54]), some simple interventions such as accuracy prompts (e.g. ‘Be sceptical of headlines. Investigate the source. Watch for unusual formatting.’) have been successful against some biases (see [55] for an intervention to reduce fake news sharing and trust in unfounded sources). Thus, we proposed that a similar approach could be useful to reduce the causal illusion. As we explained above, instructional manipulations that have been shown to reduce the causal illusion can be interpreted as debiasing interventions. In particular, Matute [31] documented a reduction in the illusion when the instructions indicated that the goal of the task was to assess causality accurately and advised participants to sample a balanced set of information (i.e. as many cause-present as cause-absent observations). Furthermore, a very similar rationale underlies several educational interventions to reduce causal illusions, but these combine not only debiasing information but also motivational elements (see [18]). Hence, we adopted this idea in experiment 2. The results of our experiment suggest that providing debiasing instructions reduced the causal illusion, but only for those participants who were more prone to this bias. A mediation analysis showed that the intervention seemed to work because those participants who were presented with the debiasing instructions aimed to activate their analytical thinking and behaviour. Therefore, they tended to use the treatment on a relatively fewer number of occasions, which in turn reduced the illusion. This is interesting as certain debiasing programmes, like accuracy prompts, are believed to work by activating an analytical mindset (rather than the intuitive style of thinking). We should generally remain cautious when interpreting mediational analyses even in experimental designs like ours, as an uncontrolled confounding factor between the mediator and the dependent variable can bias the estimation of the indirect path [56]. Still, the fact that the effect is completely mediated by the number of times the treatment was administered suggests that at least some debiasing techniques also affect the way information is collected. It also aligns with the idea that causal illusions are ‘learning effects’ that depend on the accuracy of the encoded information. However, we lack a proper test of this hypothesis given that our design just intended to detect the effectiveness of the intervention.

The fact that the debiasing intervention was only partially effective (it reduced, but not eliminated, the causal illusion) also deserves a comment. This type of partial reduction in the illusion is actually not an uncommon finding in educational interventions (see [20]). What these results indicate is that there is still room for further improvement. In this sense, it would be interesting for future research to test whether financial incentives might make a difference if they are included in combination with debiasing instructions. Perhaps the motivational effect of incentives becomes detectable if participants know exactly how they should proceed to improve their performance.

In summary, simple written instructions warning that judgements are often incorrect and explaining what to do and how to do it can be an effective complement to educational programmes aiming to reduce the use of pseudomedicines and causal illusions, particularly in situations where the development of this cognitive bias is very likely (for example, when resources are abundant). It can be an inexpensive, easy-to-implement and effective intervention.

## Data Availability

Data and materials for these experiments are openly available in the Open Science Framework [57]. Supplementary material is available online [58].
